# New Challenges in Cryopreservation: A Reproductive Perspective

**DOI:** 10.3390/ani12131598

**Published:** 2022-06-21

**Authors:** Daniela Bebbere, Sara Succu

**Affiliations:** Department of Veterinary Medicine, University of Sassari, Via Vienna 2, 07100 Sassari, Italy

Cryopreservation is a fundamental procedure to preserve the structure and function of cells and tissues by storing them at low temperatures for long periods. Developed in the early 1900s, cryopreservation maintains an essential role in the conservation of living material in different fields, including health, biodiversity conservation, and biotechnologies.

The field has evolved significantly since that time and extensive research has resulted in the development of new approaches to create an acceptable balance between the positive and negative effects of cryopreservation. Efforts have mainly attempted to limit the damage caused by exposure to low temperatures and to reduce the high financial costs. Most recent technologies have introduced new opportunities to optimize protocols and understand cell biological responses to cryopreservation.

Reproductive medicine has greatly benefited from the optimization of cryopreservation procedures, which currently enable the effective conservation of semen, oocytes, embryos, and gonadal tissues. The main applications are assisted reproduction in humans and domestic animals, and the creation of germplasm banks of wild species. With the increase in our knowledge in the field, and despite the relevant improvements in the application of cryopreservation, two key issues have emerged. On one side, a major challenge in the creation of wild species biobanks is the lack of species-specific protocols to meet the requirements of cells and tissues with diverse cryobiological properties [1]. On the other side, an increasing number of studies are suggesting possible long-term effects on gene regulation and phenotypes that are not obvious at the time of cryopreservation [2]. Such issues call for further studies to optimize protocols and to also deeply investigate the effects of cryopreservation on gene regulation.

The Special Issue “New Challenges in Cryopreservation” (https://www.mdpi.com/journal/animals/special_issues/New_Challenges_in_Cryopreservation) presents a collection of articles addressing aspects such as novel protocols to cryopreserve gametes of domestic and wild species and exploring the long-term effects of cryopreservation at the phenotypic and molecular levels from a developmental programming perspective.

In the context of creating germplasm banks of wild species, a major challenge to retaining the viability of frozen gametes and reproductive tissues is understanding and overcoming species specificities; as most cryopreservation protocols were optimized in domestic species or humans, the substantial diversity in cryobiological properties and requirements among cell types and tissues becomes an obstacle to the cryopreservation of wild species germplasm [1].

Medina-Chávez et al. [3] addressed this problem by proposing a novel approach to cryopreserve Red Deer (*Cervus elaphus hispanicus*) epididymal sperm. By comparing different freezing devices, techniques and lengths of equilibration, the authors identified the most suitable combination of experimental conditions to retain the viability of sperm samples in field conditions.

Supplementation with cryoprotective molecules is a promising approach to enhance survival and improve post-thawing gamete quality. As substantial diversity in cryobiological properties and requirements exists, studies on the effects of specific molecules in different species are needed. In the present article collection, a range of molecules were tested to optimize the cryopreservation of sperm in wild and domestic species. Supplementation with inositol, a widely used sugar-like nutrient, was seen to enhance sperm cryopreservation efficiency in the Mesopotamian Catfish (*Silurus triostegus*) [4].

Two natural antioxidants, ergothioneine and isoespintanol, exerted a cryoprotective effect when added to dog semen extender, reducing deleterious sperm alterations and oxidative stress in thawed semen [5]. Similarly, the use of trehalose disaccharide in the extender of cryopreserved semen increased the fertility of the rooster (*Gallus domesticus*) [6]. Finally, the addition of curcumin and/or its nanoparticles to semen extender in the rabbit improved the post-thaw quality via redox signaling and reducing the apoptosis process, confirming the antioxidant properties of the molecule [7].

The possible molecular mechanisms induced by exposure to cryoprotectant molecules were addressed in the work by García-Martínez and co-authors [8], who evaluated the expression of three aquaglyceroporins in in vitro-matured bovine oocytes exposed to ethylene glycol, dimethyl sulfoxide, or sucrose. They observed cryoprotectant-specific enhancement of AQP3 or AQP7 expression and hypothesized a role for these molecules in oocyte tolerance to hyperosmotic stress.

In an attempt to find alternative strategies to reduce the high costs of cryopreservation, we proposed a preliminary study on ovarian tissue lyophilization in the sheep model [9]. Lyophilization is the process of drying a frozen sample via sublimation of ice; the samples can be kept at room temperature or 4 °C, leading to enormous reductions in costs. Although widely used to preserve biomolecules and macromolecular assemblies, freeze-drying of cells and tissues is currently experimental. Using a novel device named Darya, we observed effects on tissue integrity and gene expression, and demonstrated that sheep ovarian tissue can tolerate the applied vitrification and drying protocol, paving the way for tissue freeze-drying optimization.

Using the rabbit as a model, the potential long-term effects of cryopreservation on gene regulation and phenotypes were evaluated in two different studies [10]. Garcia-Dominguez and co-authors observed that embryo vitrification affected offspring birthweight and growth performance in a sex-specific manner. Furthermore, molecular analyses revealed reprogramming of the liver proteome after the birth of vitrified embryos and changes in relation to oxidative phosphorylation and dysregulations in zinc and lipid metabolism.

Additional work by the same research group further demonstrated that early embryo vitrification and transfer induced paternally transmissible effects on the growth pattern and adult bodyweight, which seem to be non-inheritable via the female germline [11]. Such evidence provides striking examples of the complexity of the molecular mechanisms underlying embryo developmental plasticity, and calls for further studies on the possible long-term effects of cryopreservation.

Finally, Tharasait and Thuwanut [12] provide an overview of oocyte cryopreservation in domestic animals and humans, focusing on recent developments and future prospects for its optimization.

Long-term storage of cells and tissues can offer a wide range of applications, such as improving domestic animal breeding by genetic selection, contributing to the preservation of biodiversity through wildlife species conservation, and boosting in vitro biotechnology research and applications, thanks to the availability of stored gametes for in vitro embryo production. Despite its successful use in humans, cryopreservation remains an open challenge that still requires the contribution of different research fields to improve its efficiency and applicability.
